# Associations of Stressful Life Events With Diabetes Incidence in China: Insights From the China Kadoorie Biobank

**DOI:** 10.1111/1753-0407.70149

**Published:** 2025-09-01

**Authors:** Jing Qian, Huiying Cheng, Xuening Dai, Dianjianyi Sun, Pei Pei, Meng Wang, Yingjun Li

**Affiliations:** ^1^ Department of Epidemiology and Health Statistics School of Public Health, Hangzhou Medical College Hangzhou China; ^2^ Department of Epidemiology & Biostatistics School of Public Health, Peking University Haidian District Beijing China; ^3^ Peking University, Center for Public Health and Epidemic Preparedness and Response Beijing China; ^4^ Zhejiang Provincial Center for Disease Control and Prevention Hangzhou China

**Keywords:** China Kadoorie Biobank, diabetes mellitus, prevention strategies, public health, stressful life events

## Abstract

**Background:**

Limited empirical evidence exists on the link between exposure to various stressful life events (SLEs) and the heightened risk of Diabetes Mellitus (DM) within the mainland Chinese population.

**Methods:**

We conducted this prospective cohort study with 455,464 participants from the China Kadoorie Biobank (CKB); we examined associations between SLEs exposures and DM outcomes. We employed multivariable Cox proportional hazards models to estimate hazard ratios (HRs) and 95% confidence intervals (CIs), adjusting for potential confounders.

**Results:**

Over a median follow‐up of 10.1 years, 14,218 DM cases were identified. A dose–response relationship was observed between the number of SLEs, personal‐related events, and the risk of DM. The higher the number of SLEs experienced, the higher the risk of developing diabetes (HR = 1.06, 95% CI = 1.01–1.12); individuals who experienced personal‐related events had a higher risk of developing DM (HR = 1.17, 95% CI = 1.01–1.36), and those who experienced marital separation/divorce had a 53% increased risk of DM (HR = 1.53, 95% CI = 1.12–2.09). Subgroup analyses revealed effect modifications based on birth cohort, sex, and area.

**Conclusion:**

By exploring the association of multiple SLEs with the development of DM, we identified marital separation/divorce as a driver of increased DM risk.

## Graphical abstract

This study reveals a strong link between stressful life events and heightened diabetes risk in China. Personal ordeals, notably marital separation, significantly boost this risk. The impact varies by birth cohort, sex, and region, underscoring the critical role of social and psychological factors in diabetes prevention.

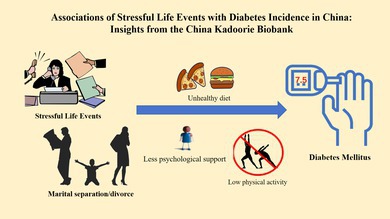

**Summary**

- This study investigates the association between stressful life events (SLEs) and the risk of developing Diabetes Mellitus (DM) in the Chinese population.
- The research involved a prospective cohort study with 455,464 participants from the China Kadoorie Biobank (CKB). The study used multivariable Cox proportional hazards models to estimate hazard ratios (HRs) and 95% confidence intervals (CIs), adjusting for potential confounders.

## 1 Introduction

Diabetes Mellitus (DM), a chronic metabolic disorder, is surging in prevalence globally, representing a formidable public health challenge. According to the latest 2024 study by the World Health Organization, the prevalence of DM among adults worldwide increased from 7% to 14% between 1990 and 2022. Currently, over 800 million adults globally are living with DM [1]. The increasing burden of this disease is concerning, making DM prevention a critical priority. While lifestyle factors such as unhealthy diet [2], physical inactivity [3], and obesity [4] are widely recognized and extensively studied contributors to DM risk, psychosocial and social support factors have garnered increasing attention in recent research, such as stress, loneliness, and social isolation [5, 6, 7, 8]. Much of the development of these psychosocial problems is attributable to the impact of stressful life events (SLEs) [9, 10, 11, 12]. SLEs are significant events or situations that negatively affect an individual's physical and mental health and are usually accompanied by strong emotional reactions and psychological stress. Several studies have been conducted to explore the relationship between some SLEs and DM. A meta‐analysis that included seven articles showed a significant association between adverse childhood experiences and an increased risk of developing type 2 diabetes mellitus (T2DM) in adulthood [13]. A Danish follow‐up study conducted from 1980 to 1996 found that parents who experienced the loss of a child had a 29% increased risk of first hospitalization for type 1 diabetes mellitus (T1DM) and a 44% increased risk for T2DM. Notably, the elevated risk of hospitalization for T2DM was statistically significant only among mothers [14]. A cohort study from the Whitehall II study in the UK revealed that the more individuals experience SLEs, the higher their risk of developing DM [15]. A study from the China Health and Retirement Longitudinal Study (CHARLS) examined the association between 12 Adverse Childhood Experiences (ACEs) and the risk of several chronic diseases among middle‐aged and older Chinese adults and found that exposure to ACEs was associated with a higher risk of several chronic diseases, including DM [16]. A retrospective cohort study from China examined the effect of early famine experience on adult‐onset DM and impaired fasting glucose in 19,347 study participants and found that early famine experience significantly increased the risk of adult‐onset DM and impaired fasting glucose in men [17]. Currently, there are limited large‐sample population‐based studies in Chinese populations, and most of them are retrospective, and the inference of causal associations needs to be improved. Furthermore, there are no studies that have comprehensively examined the association between individual‐, family‐, and work‐related SLEs and the risk of DM.

Therefore, the purpose of this paper is to use the China Kadoorie Biobank (CKB), a large prospective study cohort, to explore the association between experiencing personal, family, and work‐related SLEs and the risk of DM in the Chinese population, and to explore the possible preventive pathways of DM occurrence from the psychosocial aspect, providing important research evidence for the multi‐pathway prevention of DM.

## 2 Methods

### 2.1 Study Design and Population

This prospective cohort study utilized data from the CKB, a large‐scale, prospective cohort study launched between 2004 and 2008 to investigate the causes of chronic diseases, including cardiovascular disease, DM, cancer, and other major health conditions in China. One of the largest and most comprehensive biobanks globally, it includes data from 512,891 participants aged 30–79 years, drawn from 10 diverse regions across the country [18]. The CKB study protocol was approved by the Tropical Research Ethics Committee of the University of Oxford and the Ethical Review Committee of the Chinese Center for Disease Control and Prevention, and all participants provided written informed consent prior to participation in the study [19]. This study is reported according to the Strengthening the Guidelines for Reporting Observational Studies in Epidemiology (STROBE) (Table S1) [20].

**FIGURE 1:**
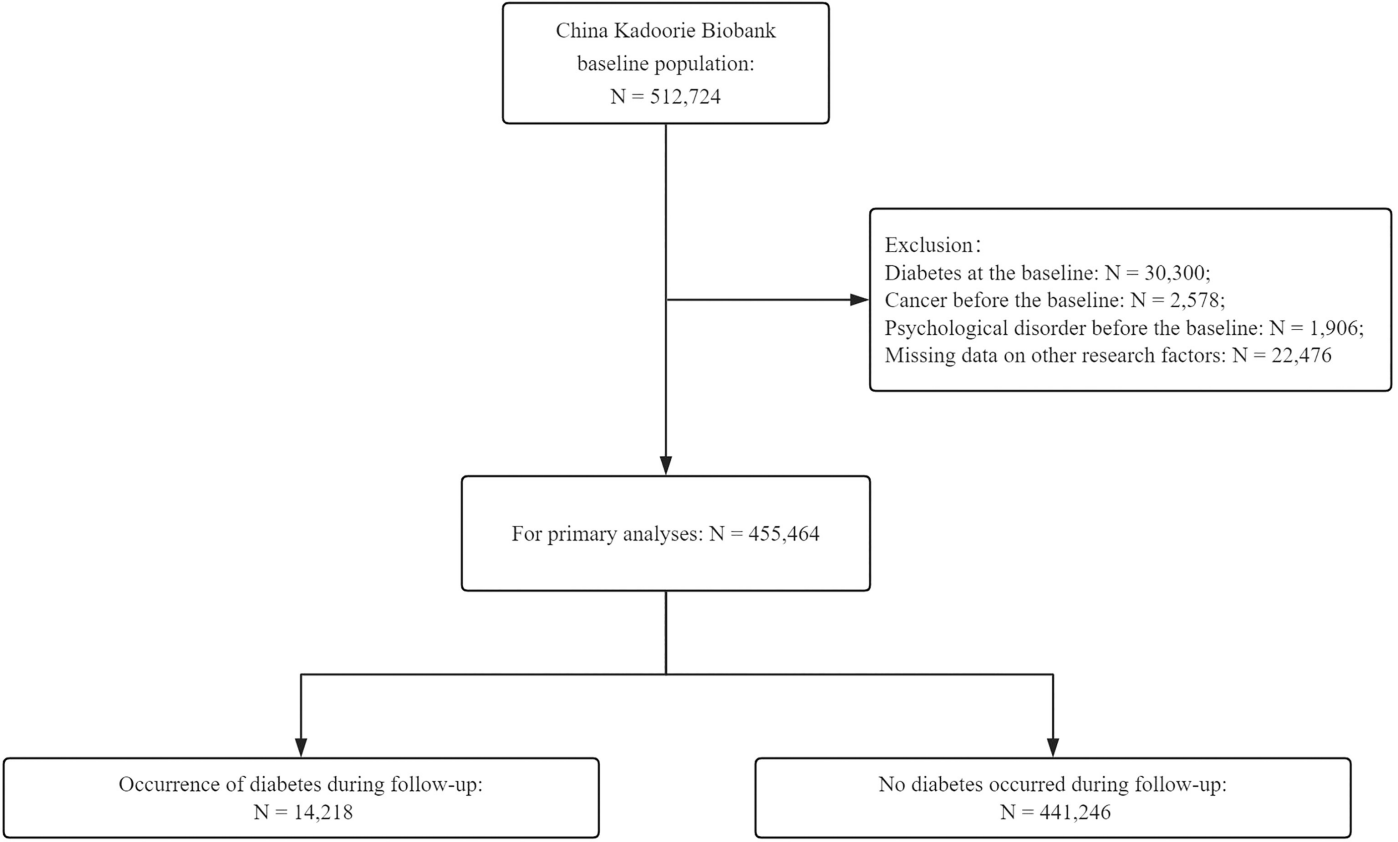
Population inclusion exclusion chart.

For this analysis, we excluded individuals with a history of DM, cancer, and psychiatric disorders at baseline. Additionally, we excluded participants with missing data on other relevant variables. The final cohort comprised 455,464 participants. A detailed flowchart of inclusion and exclusion criteria is provided in Figure 1.

### 2.2 SLEs Ascertainment

The primary exposure variable in this study was the occurrence of SLEs. Participants were queried about their experiences over the past two years with the question, “Have you encountered any of the following significant life events?” Each of the ten listed events was responded to with a binary choice of “No” or “Yes”. Utilizing the Cobb‐Clark and Schurer classifications, the ten life events were categorized in this study as follows: (1) work‐related events, including loss of job or retirement, business bankruptcy, loss of income or living on debt; (2) family‐related events, including major conflict within the family, death or major illness of spouse or other close family member; and (3) personal‐related events, including marital separation or divorce, victim of violence, major injury or traffic accident, or major natural disaster [21]. In this study, we separately analyzed the number of SLEs (the sum of the number experienced in the ten SLEs), the three types of SLEs (work‐, family‐, and personal‐related), and the association of the specific ten SLEs with DM. By examining the different categorizations of SLEs, our analyses utilized exposure information across the board.

### 2.3 DM Ascertainment

The primary outcomes were the risks of DM. Diagnoses were ascertained from inpatient medical records. Follow‐up began at baseline and ended at the first occurrence of DM, death, loss to follow‐up, or the study's end date, whichever came first. The censoring date was December 13, 2016.

### 2.4 Covariates Ascertainment

To comprehensively account for factors influencing DM risk, we included covariates spanning sociodemographic characteristics, physical health indicators, and lifestyle behaviors. The selection of covariates was guided by prior research and expert opinion [22, 23, 24]. Sociodemographic characteristics included birth cohort (1925 ~ 1939, 1940 ~ 1949, and 1950 ~ 1978), sex (male/female), area (rural/urban), education (no formal school, primary school, middle school, high school, technical school/college, and university), and household income (< 2500 yuan/year, 2500 ~ 4999 yuan/year, 5000 ~ 9999 yuan/year, 10 000 ~ 19 999 yuan/year, 20 000 ~ 34 999 yuan/year). Physical health indicators included Waist‐to‐hip ratio (WHR) (Female < 80, Male < 90; Female ≥ 80, Male ≥ 90), Body mass index (BMI) (underweight (< 18.5), normal weight (18.5 ~ 23.9), overweight (23.9 ~ 27.9), and obesity (≥ 27.9)) and Family history of DM (defined as a variable of “Yes” if any of the four categories of relatives‐father, mother, siblings, and children‐had DM; otherwise, “No”). Lifestyle behaviors included alcohol drinking (Never, Sometimes, Often), smoking frequency (Never, Sometimes, Often), metabolic equivalents of task (MET: The ratio of the rate at which a person expends energy, including a day's work and leisure activities. Use quartile grouping.) [25].

### 2.5 Statistical Analysis

We began with descriptive analyses to summarize the baseline characteristics of the study population. For categorical variables, we reported counts and percentages, comparing differences in proportions between groups using the chi‐square test. To examine the association between SLEs exposure and DM risk, we employed multivariable Cox proportional hazards models using the survival package in R. These models estimated hazard ratios (HRs) and their corresponding 95% confidence intervals (CIs), adjusting for a range of confounders. The covariates were introduced gradually, as outlined in the covariates section, including sociodemographic characteristics, physical health indicators, and lifestyle behaviors.

We conducted subgroup analyses to explore potential effect modifications based on birth cohort (1925 ~ 1939, 1940 ~ 1949, 1950 ~ 1978), sex (male/female), and area (urban/rural). In addition, we performed sensitivity analyses to assess the robustness of our findings under different assumptions and conditions: to minimize the effect of reverse causality, we excluded patients who developed DM within two years after baseline. All statistical tests were performed using R software version 4.4.1, with statistical significance set at *p* < 0.05.

## 3 Results

During a median follow‐up period of 10.1 years, 14,218 cases of DM were identified among 455,464 individuals initially free of DM (Figure 1). Cases occurred mainly in the group of women born between 1950 and 1978, in the rural population, with a low level of education, without smoking and drinking habits, overweight, and with a family history of DM. More details regarding the socio‐demographic characteristics of the participants can be found in Table 1.

**TABLE 1:** Basic characteristics and incidence of diabetes in China Kadoorie Biobank.

	Development of diabetes		*p* value
	No	Yes	
*N*	441 246	14 218	
Birth cohort(%)			< 0.001
1925 ~ 1939	45 569 (10.4)	2070 (14.6)	
1940 ~ 1949	87 552 (19.8)	4096 (28.8)	
1950 ~ 1978	308 125 (69.8)	8052 (56.6)	
Area (%)			< 0.001
Rural	251 452 (57.0)	7813 (55.0)	
Urban	189 794 (43.0)	6405 (45.0)	
Sex (%)			
Male	179 493 (40.7)	5451 (38.3)	
Female	261 753 (59.3)	8767 (61.7)	
Education (%)			< 0.001
No formal school	0 (0.0)	0 (0.0)	
Primary School	79 189 (17.9)	3530 (24.8)	
Middle School	140 681 (31.9)	5003 (35.2)	
High School	128 079 (29.1)	3225 (22.7)	
Technical school/college	67 988 (15.4)	1715 (12.1)	
University	25 309 (5.7)	745 (5.2)	
Household income (%)			< 0.001
< 2500 yuan/year	12 041 (2.7)	501 (3.5)	
2500 ~ 4999 yuan/year	29 166 (6.6)	916 (6.4)	
5000 ~ 9999 yuan/year	82 568 (18.7)	2061 (14.6)	
10 000 ~ 19 999 yuan/year	128 643 (29.2)	3662 (25.8)	
20 000 ~ 34 999 yuan/year	109 419 (24.8)	3860 (27.1)	
≥ 35 000 yuan/year	79 409 (18.0)	3218 (22.6)	
Alcohol drinking (%)			< 0.001
Never	206 475 (46.8)	7639 (53.7)	
Sometimes	150 983 (34.2)	3990 (28.1)	
Often	83 788 (19.0)	2589 (18.2)	
Smoking frequency (%)			< 0.001
Never	310 612 (70.4)	10 338 (72.7)	
Sometimes	19 729 (4.5)	603 (4.2)	
Often	110 905 (25.1)	3277 (23.1)	
Metabolic equivalents of task (%)			< 0.001
Q1	109 889 (24.9)	4055 (28.5)	
Q2	110 150 (25.0)	3686 (25.9)	
Q3	110 495 (25.0)	3345 (23.6)	
Q4	110 712 (25.1)	3132 (22.0)	
Waist‐to‐hip ratio (%)			< 0.001
Female < 80, Male < 90	294 679 (66.8)	5787 (40.7)	
Female ≥ 80, Male ≥ 90	146 567 (33.2)	8431 (59.3)	
Body mass index (%)			< 0.001
Under weight (≤ 18.5)	21 097 (4.8)	253 (1.8)	
Normal weight (18.5 ~ 23.9)	234 071 (53.0)	4102 (28.9)	
Overweight (23.9 ~ 27.9)	143 637 (32.6)	6342 (44.5)	
Obesity (≥ 28)	42 441 (9.6)	3521 (24.8)	
Family history of diabetes (%)			< 0.001
No	413 286 (93.7)	12 643 (88.9)	
Yes	27 960 (6.3)	1575 (11.1)	

**FIGURE 2:**
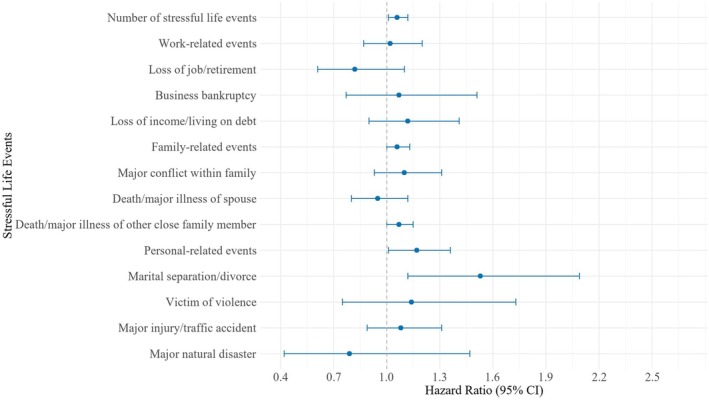
Forest plot of the associations between exposure to stressful life events and diabetes risk.

We observed a dose–response relationship between the number of SLEs, personal‐related events, and experiencing marital separation/divorce and the risk of DM. In the fully corrected model, the more SLEs people experience, the higher their risk of developing DM (HR = 1.06, 95% CI = 1.01–1.12). People who experienced personal‐related events are at a higher risk of developing DM (HR = 1.17, 95% CI = 1.01–1.36). Those who experienced marital separation/divorce had a 53% increased risk of DM (HR = 1.53, 95% CI = 1.12–2.09) (Table 2, Figure 2).

**TABLE 2:** Associations between exposure to stressful life events and diabetes risk in China Kadoorie Biobank.

Category	Cases of diabetes/Total	Model 1		Model 2		Model 3	
		HR (95% CI)	*p* value	HR (95% CI)	*p* value	HR (95% CI)	*p* value
Number of stressful life events	14 218/455464	1.05 (0.99, 1.10)	0.095	1.05 (0.99, 1.10)	0.088	1.06 (1.01, 1.12)	0.022
Specific stressful life events							
Work‐related events (Ref = No)	152/5244	1.01 (0.86, 1.18)	0.913	0.99 (0.84, 1.16)	0.908	1.02 (0.87, 1.20)	0.764
Loss of job/retirement	45/2010	0.79 (0.59, 1.07)	0.124	0.76 (0.57, 1.02)	0.071	0.82 (0.61, 1.10)	0.190
Business bankruptcy	34/1086	1.12 (0.80, 1.57)	0.515	1.12 (0.80, 1.57)	0.510	1.07 (0.77, 1.51)	0.675
Loss of income/living on debt	75/2327	1.10 (0.87, 1.38)	0.428	1.09 (0.86, 1.37)	0.476	1.12 (0.90, 1.41)	0.312
Family‐related events (Ref = No)	990/29555	1.05 (0.98, 1.12)	0.172	1.05 (0.98, 1.12)	0.141	1.06 (1.00, 1.13)	0.070
Major conflict within family	129/3888	1.02 (0.85, 1.21)	0.845	1.03 (0.86, 1.23)	0.753	1.10 (0.93, 1.31)	0.276
Death/major illness of spouse	141/4007	0.96 (0.81, 1.13)	0.595	0.95 (0.80, 1.12)	0.529	0.95 (0.80, 1.12)	0.547
Death/major illness of other close family member	746/22419	1.06 (0.99, 1.15)	0.098	1.07 (0.99, 1.15)	0.076	1.07 (1.00, 1.15)	0.064
Personal‐related events (Ref = No)	172/5091	1.13 (0.98, 1.32)	0.102	1.14 (0.98, 1.33)	0.084	1.17 (1.01, 1.36)	0.042
Marital separation/divorce	40/1163	1.43 (1.05, 1.96)	0.025	1.44 (1.06, 1.97)	0.022	1.53 (1.12, 2.09)	0.008
Victim of violence	22/633	1.10 (0.72, 1.68)	0.649	1.11 (0.73, 1.69)	0.621	1.14 (0.75, 1.73)	0.546
Major injury/traffic accident	100/2940	1.07 (0.88, 1.30)	0.514	1.07 (0.88, 1.31)	0.486	1.08 (0.89, 1.31)	0.448
Major natural disaster	10/433	0.77 (0.41, 1.43)	0.402	0.78 (0.42, 1.46)	0.438	0.79 (0.42, 1.47)	0.457

*Note:* Model 1 adjusted for birth cohort, sex, area, education, household income; Model 2 adjusted for Model 1 plus Smoking, Alcohol drinking, Physical activity (Metabolic Equivalents of Task, h/d); Model 3 adjusted for Model 2 plus body mass index, waist circumference, family history of diabetes; The number of stressful life events ranged from 0 to 6.

Abbreviations: 95% CI, 95% confidence interval; HR, hazard ratio.

In results stratified by age, individuals born in 1950–1978 had a significantly increased risk of DM after experiencing multiple SLEs (HR = 1.09, 95% CI = 1.02–1.17, *p* = 0.014), as well as marital separation/divorce (HR = 1.63, 95% CI = 1.16–2.29, *p* = 0.005). Although the interaction *p* values for age group comparisons were not statistically significant (*p* for interaction = 0.332 for number of SLEs and *p* for interaction = 0.944 for marital separation/divorce), the effect appears more pronounced in this younger cohort (Table S2). In analyses stratified by sex, women demonstrated a significantly increased risk of DM after exposure to multiple SLEs (HR = 1.10, 95% CI = 1.04–1.18, *p* = 0.002), family‐related events (HR = 1.09, 95% CI = 1.01–1.18, *p* = 0.031), and personal‐related events (HR = 1.22, 95% CI = 1.01–1.48, *p* = 0.040). The interaction *p* value for number of SLEs was statistically significant (*p* for interaction = 0.018), indicating a stronger overall effect in females compared to males. Conversely, in men, marital separation/divorce conferred a higher risk (HR = 1.77, 95% CI = 1.13–2.79, *p* = 0.013), although the interaction between sex and this event was not statistically significant (*p* for interaction = 0.598) (Table S3). In results stratified by area, urban residents who experienced personal‐related SLEs were at significantly higher risk of DM (HR = 1.36, 95% CI = 1.12–1.67, *p* = 0.002), with a statistically significant interaction by area (*p* for interaction = 0.021). Additionally, a reduced risk of DM was observed in urban participants who experienced loss of job or retirement (HR = 0.67, 95% CI = 0.48–0.92, *p* = 0.014), though the interaction test was not statistically significant (*p* for interaction = 0.255) (Table S4). Sensitivity analyses (excluding DM onset diagnoses within two years of baseline) confirmed the robustness of our results (Table S5).

## 4 Discussion

This study highlights the impact of SLEs exposure on DM risk within the CKB cohort. Our findings indicate that an increase in SLEs within a population is associated with a heightened risk of developing DM. Specifically, personal‐related SLEs—such as marital separation/divorce, experiences of violence, major injuries/traffic accidents, and natural disasters—are strongly linked to an elevated DM risk. Among these, marital separation/divorce emerged as the most impactful SLE contributing to this increased risk and was particularly significant in men.

Marital separation/divorce often leads to substantial socio‐economic shifts, potentially disrupting access to economic resources and altering social status. These changes can be linked to a heightened risk of experiencing negative health consequences [26]. A study in the realm of divorce and health has unveiled key insights, concluding that individuals who undergo separation or divorce face an increased risk of negative health outcomes. Notably, the research indicates that the mortality rate among those who have experienced a divorce or separation is 23% higher than that of the general population [27]. A retrospective case–control study in India found a higher prevalence of DM among widowed/divorced/separated individuals [28].

Our study has revealed that younger people are more likely to develop DM after negative life events (especially marital separation/divorce) relative to older people. Younger people are more likely to develop DM after SLEs (especially separation and divorce) relative to older people. This may be due to the fact that younger people may not be as physically and psychologically resilient to negative life events as older people. Older people may have better coping mechanisms and mental toughness because they have experienced more life events. Young people may be more sensitive to the psychological impact of negative life events. Prolonged psychological stress and stress reactions may lead to changes in hormone levels, affecting insulin secretion and action and increasing the risk of DM [29]. We have also found that men are considerably more likely to develop DM following divorce or separation compared to women. This heightened risk could stem from the increased social isolation and diminished physical activity that men often encounter post‐divorce, which can precipitate unhealthy lifestyle choices such as overeating [30]. Consequently, this behavior escalates the risk of obesity and, by extension, DM. Furthermore, married men typically benefit from the dietary control and health oversight provided by their spouses, a support system that may falter or disappear after divorce, potentially leading to the adoption of detrimental eating and lifestyle habits [31]. A UK‐based longitudinal study on aging has revealed that marriage or cohabitation is associated with a reduced risk of developing T2DM. The research indicates that married individuals exhibit a 0.21% decrease in glycosylated hemoglobin levels compared to those who are single, a difference that corresponds to a significant 25% reduction in mortality risk [32]. The effect of personal‐related events on the risk of developing DM is more pronounced in urban populations. The impact of personal‐related events on the risk of developing DM is particularly pronounced among urban populations. This heightened vulnerability may stem from urban dwellers' increased susceptibility to socio‐economic stressors, which can be exacerbated following negative personal‐related events, thereby amplifying the risk of DM. Additionally, urban residents are more prone to overweight and obesity due to shifts in dietary habits and reduced physical activity, further contributing to an elevated risk of DM. We also found that urban populations experiencing loss of job/retirement have a reduced risk of developing diabetes, which may be related to the high stress of urban work that may lead to unhealthy lifestyle choices such as increased intake of high‐sugar and high‐fat foods and alcohol consumption. People with stressful jobs may be less likely to engage in physical activity, leading to weight gain and obesity, which are risk factors for diabetes, whereas the status of being unemployed or retired may give people more time to focus on their health.

The strengths of this study are its large cohort and long follow‐up period, which increases the reliability of the findings. Moreover, this study comprehensively explored the association between the three main types and ten specific types of SLEs and the development of DM, enriching the results of this research theme. However, our study also has some limitations. Firstly, while the analysis took into account a fairly comprehensive array of potential confounding factors, the possibility of residual confounding due to unmeasured variables cannot be entirely ruled out, such as dietary factors, which have an important role in the development of DM; therefore, dietary factors should be included in subsequent studies to analyze them as well, in order to correct for confounding factors more comprehensively. Secondly, our study population was limited to Chinese populations and cannot be extrapolated to other ethnic groups. Thirdly, the stressful life events examined in this paper relied on self‐reporting, and thus bias may have arisen during the collection process, affecting the reliability of the observations in this study. Future studies should focus on conducting more comprehensive analyses to better understand the potential link between SLEs and the development of DM [33].

## 5 Conclusion

This study delves into the association between SLEs and the development of DM, revealing the potential impact of major life stresses and traumas on an individual's risk of DM. The findings highlight the importance of raising public health awareness, guiding prevention strategies, optimizing individualized treatment plans, and strengthening social support networks. These findings are of great practical significance for reducing DM risk and improving patient self‐management, and provide a scientific basis for public health policy makers. In summary, this study not only provides new perspectives on DM prevention and intervention, but also lays the foundation for interdisciplinary cooperation and the development of integrated management strategies.

## Conflicts of Interest

The authors declare no conflicts of interest.

## Supporting information


**Data S1:** Supporting Information.
